# A retrospective analysis of weight changes in HIV-positive patients switching from a tenofovir disoproxil fumarate (TDF)- to a tenofovir alafenamide fumarate (TAF)-containing treatment regimen in one German university hospital in 2015–2017

**DOI:** 10.1007/s15010-018-1227-0

**Published:** 2018-09-29

**Authors:** Mario Gomez, Ulrich Seybold, Julia Roider, Georg Härter, Johannes R. Bogner

**Affiliations:** 1 Sektion Klinische Infektiologie, Medizinische Klinik und Poliklinik IV, Klinikum der Universität, Ludwig-Maximilians-Universität München, Pettenkoferstrasse 8a, 80336 Munich, Germany; 2 Medicover Ulm MVZ, Münsterplatz 6, 89073 Ulm, Germany

**Keywords:** Tenofovir disoproxil fumarate (TDF), Tenofovir alafenamide fumarate (TAF), Weight, Retrospective cohort study

## Abstract

**Purpose:**

To determine whether changing from a tenofovir disoproxil fumarate (TDF)- to a tenofovir alafenamide fumarate (TAF)-containing regimen is correlated with weight changes in a human immunodeficiency virus (HIV)-positive adult cohort.

**Methods:**

Retrospective analysis was conducted of data gathered from routine care in a university hospital in Munich, Germany, between July 2015 and June 2017. Data from patients’ charts were extracted and a two-step approach was applied. First, weight/BMI progression within 1 year after initiation of either TDF or TAF was compared. Subsequently, weight measurements within subjects changing from a TDF- to a TAF-containing antiretroviral regimen were analyzed by means of a repeated measurements general linear model.

**Results:**

After 360 days of initiating TAF, patients showed a mean (± standard deviation) percentual weight increase of 3.17 ± 0.21, whereas after 360 days of initiating TDF, patients only showed a mean (± standard deviation) percentual weight increase of 0.55 ± 0.17. The repeated measurements general linear model for within-subjects design showed a statistically significant correlation in weight after changing from a TDF to a TAF containing antiretroviral regimen. The weight difference between the two measurements while on TDF was not statistically significant, but every measure after switching to TAF was significantly higher than the previous.

**Conclusion:**

Changing from a TDF- to a TAF-containing regimen is correlated with weight gain in this retrospectively analyzed real-world cohort in Munich, Germany.

## Introduction

Tenofovir alafenamide fumarate (TAF) is a prodrug of tenofovir, identified as an alternative to tenofovir disoproxil fumarate (TDF) in 2004, because of its preferential distribution into peripheral blood mononuclear cells (PBMCs), achieving considerably higher concentrations of tenofovir inside PBMCs with a much lower loading dose [1]. It was first approved for use in the European Union in November 2015 as a constitutive component of a single-tablet regimen (STR) with elvitegravir, cobicistat, and emtricitabine [2]. The non-inferiority of TAF versus TDF in terms of virologic efficacy has been demonstrated in two double-blind Phase 3 studies [3]. Also, an improved bone and kidney safety profile [4] has been found, independent of the third compound [5]. In the meantime, further STRs have been approved in which TDF was replaced by TAF. This was done based on non-inferiority studies [6]. Real-world evidence (RWE) studies support the virologic superiority or non-inferiority of TAF [7–9]; the preservation of kidney function and the improvement or non-deterioration of bone density parameters are now widely accepted facts [10–12].

While the DHHS (U.S. Department of Health and Human Services) Guidelines [11] and the EACS (European AIDS Clinical Society) Guidelines [10] (as of September 2018) still include TDF in the recommended regimens, the IAS-USA (International Antiviral Society—USA) Guidelines [12] (last updated in 2018) recommend only TAF-containing backbones.

Retrospective cohort studies carried out in Brazil, the USA and France support the notion that antiretroviral therapies, especially strand transfer inhibitor-based regimens, are associated with weight increase [13–15]; there are, however, no published data on weight changes associated with backbone switching from a TDF- to TAF-based regimen. Unstructured, unpublished clinical observations (personal communications by colleagues/patients) indicate a potential weight gain in patients following a switch from TDF- to TAF-containing regimens. Therefore, a systematic evaluation of weight data in switch patients from the Section Clinical Infectious Diseases of the University Hospital at Ludwig-Maximilians-Universität (LMU) Munich was carried out.

In this retrospective study, we determine whether changing from a tenofovir disoproxil fumarate (TDF)- to a tenofovir alafenamide fumarate (TAF)-containing antiretroviral regimen is correlated with weight changes in a human immunodeficiency virus (HIV)-positive adult cohort, during a period of 2 years in a tertiary health care institution in Munich, Germany.

## Patients and methods

Data originated from a single center (Division of Clinical Infectious Diseases, University Hospital LMU Munich). Data from routine care patient charts from July 2015 through June 2017 were extracted and retrospectively analyzed. All available patient charts in the Clinical Infectious Diseases outpatient clinic were reviewed for pertaining general inclusion criteria. These included: HIV infection, being over 18 years of age, and having been in treatment in the clinic during the study period with either TDF- or TAF-containing regimens. For the weight analysis, weight inclusion criteria had to be met: having at least one measurement on TDF treatment and one measurement on TAF treatment (after switch) during the study period. An additional cohort of TDF-only patients with at least two weight measurements during the study period was included for reference. Patients’ charts missing important information (duration of infection, duration of treatment, nadir of TCD4 + cell count, viral load below the limit of quantification, weight measurements as described) were excluded.

Data were extracted onto an Excel Spreadsheet and all further analyses were carried out using IBM SPSS Statistics Version 24. Data were extracted from patient charts after approval from and in accordance with all applicable local ethics committee regulations.

### Weight/BMI progression within 1 year after initiation of either TAF or TDF separately using interpolation

Data were first analyzed for weight progression on TDF or TAF separately. Given that dates for weight measurements differed markedly between subjects, weight was interpolated from the available data (linear interpolation for missing values). All patients who switched from TDF to TAF had their switch date set as day zero, and positive 30-day intervals were created until day 360 (extrapolation was avoided). For TDF-only patients (and switch patients disregarding all measurements after switch), the date of the first weight measurement on TDF was set as day zero and followed the aforementioned 30-day interval logic. For separate analysis on weight progression, the data was analyzed until day 360 (day zero and 12 positive 30-days timepoints, where data were available).

### Weight/BMI changes in repeated measures general linear model (within subjects) and paired samples *t* tests

Statistical analyses were carried out to compare weight changes before and after switch (within-subjects comparison for statistical difference in weight before and after switch). For the purpose of this analysis, the first recorded weight measurement and the last recorded weight measurement while receiving TDF were used, as well as the first and last weight measurements recorded while receiving TAF. All variables were analyzed in kilograms and body mass index (BMI) scores separately. For this within-subject analysis, paired samples *t* tests and repeated measures general linear models were carried out.

For the repeated measures general lineal model, the following parameters were used: within-subject factor name “timepoint” with 4 levels for the independent variable and measure name “weight” for the dependent variable. After definition, within-subjects variables “timepoint” were defined for the levels as follows (in respective order): first weight on TDF, last weight on TDF, first weight on TAF and last weight on TAF. No covariables were defined. Where the condition of sphericity is not met (Mauchly’s test of sphericity), the Greenhouse–Geisser correction for tests of within-subjects’ effects will be interpreted. For significant tests of within-subjects’ effects, pairwise comparisons will be interpreted on the “timepoint” plot (horizontal axis defined factor “timepoint”).

All subsequent repeated measures general linear models for subpopulations follow the same operational logic described above.

## Results

From July 2015 through June 2017, 310 patients were switched from a TDF- to a TAF-containing regimen. Of the 310 patients that switched within the time period, 129 patients’ charts met the inclusion criteria and were used for analysis. In the same time period, 711 patients were treated in the clinic with a TDF-containing regimen without switch. Of the 711 patients in the TDF-only reference pool, 112 patients’ charts met the inclusion criteria and were used for analysis. Most of the patients’ charts that were excluded did not meet the criteria regarding the amount of weight measurements. Baseline characteristics for the included study population can be found in Table 1.

**Table 1:** Descriptive baseline demographics for study population at first weight measurement

	Total (*n* = 241)	Patients who changed from TDF to TAF (*n* = 129)	Patients who remained on TDF (*n* = 112)	*p* value
Age in years, mean (SD)	45.8 (11.2)	46.2 (11.5)	45.3 (10.9)	0.515
Sex				
Male, *n* (%)	177 (73.4)	105 (81.4)	72 (64.3)	0.003*
Female, *n* (%)	64 (26.6)	24 (18.6)	40 (35.7)	0.003*
Race/ethnicity				
Caucasian, *n* (%)	177 (73.4)	101 (78.3)	76 (67.9)	0.132
Black, *n* (%)	34 (14.1)	14 (10.9)	20 (17.9)	0.132
Asian, *n* (%)	21 (8.7)	8 (6.2)	13 (11.6)	0.132
Hispanic, *n* (%)	9 (3.7)	6 (4.7)	3 (2.7)	0.132
Height (m), mean (SD)	1.74 (0.10)	1.76 (0.09)	1.72 (0.10)	0.007*
Weight in (kg), mean (SD)	75.74 (15.61)	77.28 (15.04)	73.97 (16.13)	0.101
Body mass index in kg/m^2^, mean (SD)	24.92 (4.11)	25.01 (4.16)	24.82 (4.06)	0.729
Duration of HIV infection in years, mean (SD)	11.5 (8.0)	10.5 (7.7)	12.6 (8.2)	0.043*
Duration of HIV treatment in years, mean (SD)	9.0 (5.8)	8.3 (5.4)	9.8 (6.1)	0.052
Years on TDF containing regimen, mean (SD)	4.84 (3.12)	4.85 (3.04)	4.83 (3.22)	0.956
Nadir of TCD4 + cell count in cells/µL, mean (SD)	227.7 (170.9)	226.5 (173.0)	229.2 (169.0)	0.903
TCD4 + cell count over 200 cells/µL				
Yes, *n* (%)	237 (98.3)	128 (99.2)	109 (97.3)	0.249
No, *n* (%)	4 (1.7)	1 (0.8)	3 (2.7)	0.249
Viral load below limit of quantification				
Yes, *n* (%)	235 (97.9)	127 (98.4)	108 (97.3)	0.540
No, *n* (%)	5 (2.1)	2 (1.6)	3 (2.7)	0.540
Smoking status				
Never smoker, *n* (%)	85 (35.3)	41 (31.8)	44 (39.3)	0.106
Current smoker, *n* (%)	76 (31.5)	46 (35.7)	30 (26.8)	0.106
Former smoker, *n* (%)	32 (13.3)	22 (17.1)	10 (8.9)	0.106
Not reported, *n* (%)	48 (19.9)	20 (15.5)	28 (25.0)	0.106
Third agent class at baseline				
NNRTI, *n* (%)	108 (44.8)	52 (40.3)	56 (50)	0.001*
INI, *n* (%)	91 (37.8)	62 (48.1)	27 (24.1)	0.001*
PI, *n* (%)	42 (17.4)	15 (11.6)	29 (25.9)	0.001*
Third agent class after change				
NNRTI, *n* (%)		40 (31.0)		
INI, *n* (%)		81 (62.8)		
PI, *n* (%)		8 (6.2)		

*TAF* tenofovir alafenamide fumarate, *TDF* tenofovir disoproxil fumarate, *SD* standard deviation, *HIV* human immunodeficiency virus, *NNRTI* non-nucleoside reverse-transcriptase inhibitor, *INI* integrase inhibitor, *PI* protease inhibitor

*Indicates a significant *p* value

The baseline characteristics differed between the two comparison groups (patients that changed from TDF to TAF versus reference patients that remained on TDF) in some defined variables. The group of switch patients had a higher proportion of males, were slightly taller and had a shorter duration of HIV infection. Further baseline demographics such as general health status, major adverse cardiac events, malignancies, kidney diseases, thyroid dysfunction, former surgeries or treatments or physical activity were not systematically extracted out of patient charts.

An important statistical difference in baseline demographics is the distribution of patients regarding third agent class at baseline. In the group of switch patients, more were receiving an integrase inhibitor at baseline in comparison to patients who were not switched. Table 2 further describes the third agent class regimen changes made by patients in the TDF to TAF switch group.

**Table 2:** Third agent class switch in patients within the TDF to TAF switch group

	Third class agent at baseline →	NNRTI (*n* = 52)	INI (*n* = 62)	PI (*n* = 15)
Third class agent after switch →	NNRTI	39 (75%)	**1 (2%)**	0
	INI	**13 (25%)**	61 (98%)	**7 (46%)**
	PI	0	0	8 (54%)

Changes to another third agent class are indicated by bold numbers

In absolute numbers, for patients that were switched from TDF to TAF (*n* = 129) at any given point of time within the observation period, 70% showed a weight increase between the first weight measurement on TDF and the last weight measurement on TAF, while 21% of the patients showed a weight loss. 9% showed no weight change Among patients with a weight gain, 33% showed an increase of ≤ 3% of their body weight compared to baseline, 48% showed an increase of ≥ 3% to ≤ 10%, and 19% showed an increase of ≥ 10% compared to baseline.

The first weight measurement on TDF was performed 302 ± 122 days (mean ± standard deviation) before switching to TAF. The last weight measurement on TDF was performed 33 ± 78 days before switching to TAF. The first weight measurement on TAF was performed 115 ± 60 days after switching to TAF. The last weight measurement on TAF was performed 273 ± 102 days after switching to TAF. The median number of weight measurements was 4 (interquartile range, IQR 3–5) on TDF (*n* = 241) and 3 (IQR 2–3) on TAF (*n* = 129).

### Weight progression within 1 year after initiation of either TAF or TDF

**Fig. 1:**
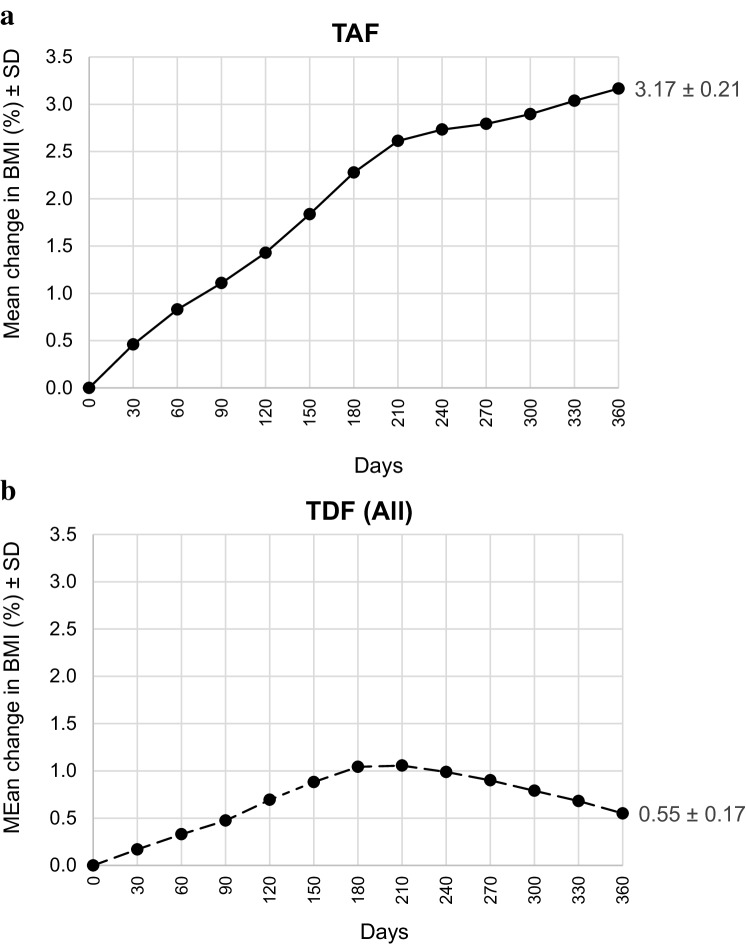
Mean change in weight in percent ± standard deviation through 360 days for TDF and TAF separately; **a** results of patients after switch to TAF (*n* = 129) and **b** results of patients receiving TDF; this arm comprises 241 patients, since switch patients initially received TDF (pooled data from the switch group and control group)

A plot of percentage weight progression through 360 days after the first measurement on TDF and TAF is shown in Fig. 1.

Table 3 shows the number of patients contributing to each timepoint for Fig. 1a and b.

**Table 3:** Number of patients assessed for each timepoint through 360 days

Timepoint →	0	30	60	90	120	150	180	210	240	270	300	330	360
TAF →	129	128	126	125	118	110	105	92	85	75	55	44	27
TDF →	241	230	230	226	214	213	204	186	182	167	151	144	129

For assessment, interpolated data were used

Patients who were switched to TAF showed a mean increase in weight of 3.17% through day 360 after change, while patients on a TDF-containing regimen showed a mean increase in weight of only 0.55% during the same 360 days window.

### Weight changes in repeated measures general linear model (within subjects) and paired samples *t* tests

**Fig. 2:**
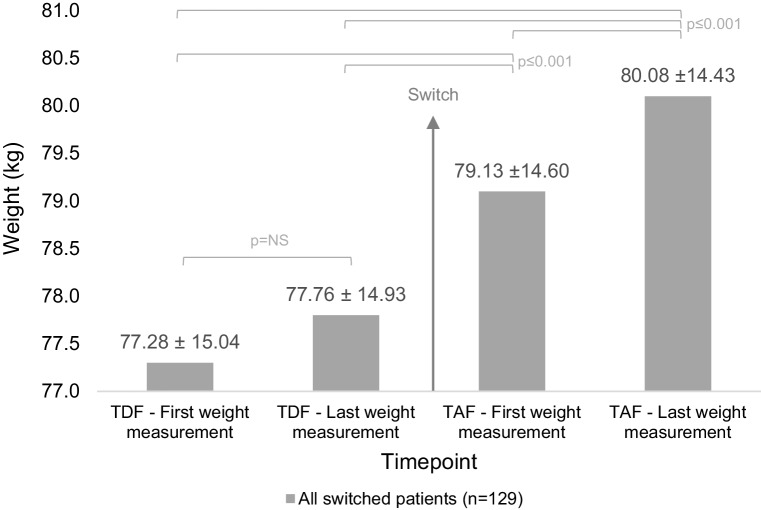
Estimated marginal means (± standard deviation) using the repeated measurements general linear model; only switch patients are shown, “TDF (TAF)—first/last weight measurement” denotes the first/last weight measured on TDF (TAF) treatment; results shown for weight in kg; *NS* not statistically significant

For switch patients, outcomes of the repeated measurements general linear model using weight measurements at four timepoints (first and last on TDF and first and last on TAF) are shown in Fig. 2.

For analysis based on weight in kilograms, the results of the repeated measurements general linear model showed that there was a significant main effect of timepoint (being on TDF or after switch to TAF) on the weight of patients (Greenhouse–Geisser correction F(1.991, 254.795) = 25.947, *p* < 0.001, *η*_*p*_^2^ = 0.169). Post hoc tests using the Bonferroni correction showed that patients had a significantly higher weight in the first TAF weight measurement (mean = 79.13; SD = 14.60) compared to the last TDF weight measurement (mean = 77.76; SD = 14.93, *p* = 0.001) and compared to the first TDF weight measurement (mean = 77.28; SD = 15.04, *p* < 0.001). Likewise, patients had a significantly higher weight in the last TAF weight measurement (mean = 80.08; SD = 14.43) compared to the last TDF weight measurement (mean = 77.76; SD = 14.93, *p* < 0.001) and compared to the first TDF weight measurement (mean = 77.28; SD = 15.04, *p* < 0.001). Furthermore, patients had a significantly higher weight in the last TAF weight measurement (mean = 80.08; SD = 14.43) compared to the first TAF weight measurement (mean = 79.13; SD = 14.60, *p* = 0.001). However, there was no statistical significance in the difference of the last TDF weight measurement (mean = 77.76; SD = 14.93) compared to the first TDF weight measurement (mean = 77.28; SD = 15.04, *p* = 0.442).

For analysis based on BMI, the results showed the same statistical significance for all timepoints.

Individual paired samples *t* tests for first and last TDF weight measurements, both in BMI in kg/m^2^ and weight in kilograms, were carried out and corroborated the non-significance in weight changes between TDF weight measurements’ timepoints. Comparisons between first and last TDF weight measurements by subgroup (TDF-only reference group, *n* = 112, and switch patients before switch, *n* = 129) were also non-significant.

Sensitivity analyses for weight changes were carried out in terms of third agent class after change. Of the 129 switched patients, 108 did not change third agent class in the observation period. Within this subpopulation, the results were comparable to those of the complete switch group: statistical significance was reached for all TAF timepoints compared to all TDF timepoints (and the last TAF over the first TAF timepoint) and the non-significant difference between TDF weight measurements also remained.

For the inverse case, patients that did change third agent class (*n* = 21), only the last weight measurement on TAF was statistically higher than the first weight measurement on TDF; no other significant differences were observed. The absolute difference between the last weight measurement on TAF and the first weight measurement on TDF was also the lowest for this subgroup (in comparison to no-switch patients and overall study population).

Table 4 shows weight at the four defined timepoints for the complete switch group and the subgroups of switch/no-switch of third agent class.

**Table 4:** Mean and standard deviation of weight measurements for sensitivity analysis of third agent class after change

	All TDF to TAF switch (*n* = 129)	Third agent class no switch (*n* = 108)	Third agent class switch (*n* = 21)
TDF—first weight measurement, mean (SD)	77.28 ± 15.04	76.94 ± 15.41	78.99 ± 13.19
TDF—last weight measurement, mean (SD)	77.76 ± 14.93	77.36 ± 15.29	79.87 ± 13.00
TAF—first weight measurement, mean (SD)	79.13 ± 14.60	78.82 ± 14.87	80.69 ± 13.28
TAF—last weight measurement, mean (SD)	80.08 ± 14.43	79.80 ± 14.78	81.53 ± 12.69

## Discussion

Switching from a TDF- to a TAF-containing regimen for the treatment of HIV is correlated with a weight increase in this retrospective, monocentric real-world cohort of adult HIV-positive patients followed for up to 2 years. While weight does not statistically increase between measurements on a TDF-containing regimen (in kilograms: 77.28–77.76, *p* = 0.442), switching to a TAF-containing regimen is associated with a statistically significant higher weight for the first weight measurement on TAF (for the last TDF to the first TAF weight measurement: 77.76–79.13, *p* = 0.001. Furthermore, the last weight measurement on TAF is also significantly higher than the first weight measurement on TAF (in kilograms: 79.13–80.08, *p* = 0.001) (and all TDF measurements). The fact that weight does not change on TDF treatment but steadily increases as shown by repeated weight measurements on TAF treatment supports the notion that TAF is correlated with weight gain after switching from TDF. The clinical relevance lies in the unclear impact of the resulting weight increase in general health parameters such as metabolic profile and blood pressure (among others). Furthermore, patients noticing weight changes could ask for a regimen change, which is thus far not recommended in this context for HIV patients.

Patients assessed in this retrospective cohort had been on a stable TDF-containing regimen for 4.84 ± 3.12 years, leveling out (but not ruling out) possible confounders related to prior history of no TDF regimen or sequential regimen changes leading to possible weight changes. While the first weight measurement on TDF dates almost a year before the switch to TAF, the first measurement on TAF occurred at approximately 4 months after switch and the last measurement approximately 9 months after the switch to TAF, giving time for weight changes to be noticeable.

Literature supporting the hypothesis of weight gain on antiretroviral regimen is scarce, but growing. A retrospective analysis from a Brazilian cohort (*n* = 1794) identified factors associated with obesity after initiation of antiretroviral regimen including not only traditional factors such as female sex, but also HIV-specific factors such as more advanced HIV disease and use of integrase inhibitors [13]. A retrospective observational cohort study carried out in the USA (*n* = 495) assessed weight changes when switching from an efavirenz-based to an integrase inhibitor-based antiretroviral regimen over 18 months. Of the 136 patients that switched, a weight gain of 2.9 kg at month 18 was observed, compared to 0.9 kg for patients that remained on the efavirenz-based regimen. Of all integrase inhibitor-based antiretroviral therapies, patients that switched to a dolutegravir-based regimen showed the greatest weight gain (5.3 kg after 18 months compared to the efavirenz-based regimen) [14]. A retrospective analysis of a real-life cohort in France (*n* = 462) found, for patients initiating a dolutegravir-based combined antiretroviral regimen, a mean weight gain of 3 kg and a mean BMI increase of 1 kg/m^2^ point after approximately 9 months (± 79 days) [15]. The results from the two latter analyses show great similarity with the results found for the cohort analyzed in this paper: weight increase of 2.32 kg (or 0.78 kg/m^2^ BMI) from the last measurement before switch (33 ± 78 days before switching to TAF) to the last measurement after switch (273 ± 102 days after switching to TAF); this is, approximately 1 month before switch to approximately 9 months after switch. However, the results from the aforementioned cohorts showed weight gains associated with switching to or initiating an integrase inhibitor-based antiretroviral regimen; this association could not be confirmed in our cohort, given that the subpopulation of patients switching to an INI-based regimen (parallel to switching from a TDF- to a TAF-based regimen) showed a lower absolute weight gain when compared to patients who did not change third agent class or all switch patients. However, the number of patients in this subgroup was very small so that a correlation cannot be ruled out.

The main limitation of this study lies in its retrospective design; retrospective data analysis can only measure correlation, but no causality. A monocentric study design cannot exclude patient-clinic preferences; the data were collected at a tertiary care institution in a major German city, therefore the patients included could exhibit certain specific characteristics inherent to those consulting such centers, e.g., higher complexity of patients’ disease status. Moreover, possible confounders like smoking, exercise and eating habits are not controlled in a real-world setting and could majorly influence weight. However, the cohort is deemed to be representative for the local setting.

Smoking is a possible bias requiring further investigation in a prospective study setting. It is well known that smoking cessation leads to weight increases similar to those seen in this cohort [16]; however, the patients’ charts did not allow for extraction of valid data on smoking cessation or even accurate smoking (change) attitudes. Additionally, the number of patients whose smoking habits was not reported (15.5%-25%) would have biased a possible analysis based on this parameter for this cohort. The heterogeneity in smoking status, nonetheless, would be expected to have a minor impact on weight for this cohort.

There was also no clear systematic in weight recording among patients, so that the lack of weight measurements in some patients’ charts could have been associated with their weight progression (or lack thereof). Forty-two percent of patients switched in the clinic during the study period met the inclusion criteria and were assessed in this study. It remains impossible to rule out that for the remaining 58% of patients with no documented weight measurements, weight was not assessed because no weight gain was subjectively observed. This would overestimate the results of this study. Additionally, only 16% of all patients remaining on TDF met the inclusion criteria and were assessed, so a majority of patients that did not switch could not be assessed. This poses a possible bias that the retrospective design of this study cannot elucidate.

The reason for switching from TDF to TAF in the evaluated patients is one important question that remains unanswered. As noted previously, TAF was first approved for use in the German setting in November 2015 as part of a single-tablet regimen, so that the high number of patients changed from TDF to TAF over the following months can be attributed to the availability of the drug, permitting simplification of regimen at the time. Nevertheless, the actual reasons have not been documented for this study.

In conclusion, these data suggest that weight could be influenced when changing HIV-treating regimens in the general HIV-infected population; the pathophysiological mechanism remains unclear. With increasing efficacy and safety profiles of treatments, health concerns shift to other aspects of patients with HIV infection that pertain to their long-term health. Prospective, controlled studies are necessary to establish causality and systematically control for known and unidentified confounders.
