# Correction to: A retrospective analysis of weight changes in HIV-positive patients switching from a tenofovir disoproxil fumarate (TDF)- to a tenofovir alafenamide fumarate (TAF)-containing treatment regimen in one German university hospital in 2015–2017

**DOI:** 10.1007/s15010-018-1251-0

**Published:** 2018-11-19

**Authors:** Mario Gomez, Ulrich Seybold, Julia Roider, Georg Härter, Johannes R. Bogner

**Affiliations:** 1 Sektion Klinische Infektiologie, Medizinische Klinik und Poliklinik IV, Klinikum der Universität, Ludwig-Maximilians-Universität München, Pettenkoferstrasse 8a, 80336 Munich, Germany; 2 Medicover Ulm MVZ, Münsterplatz 6, 89073 Ulm, Germany

## Correction to: Infection 10.1007/s15010-018-1227-0

The original version of this article unfortunately contained mistakes. The presentation of Fig. 1 was incorrect and the Acknowledgements were missing. Please find the Acknowledgement here:

Acknowledgement

This work was partly supported by DZIF project Tl 02.001.

The corrected figure is given below.

**Fig. 1:**
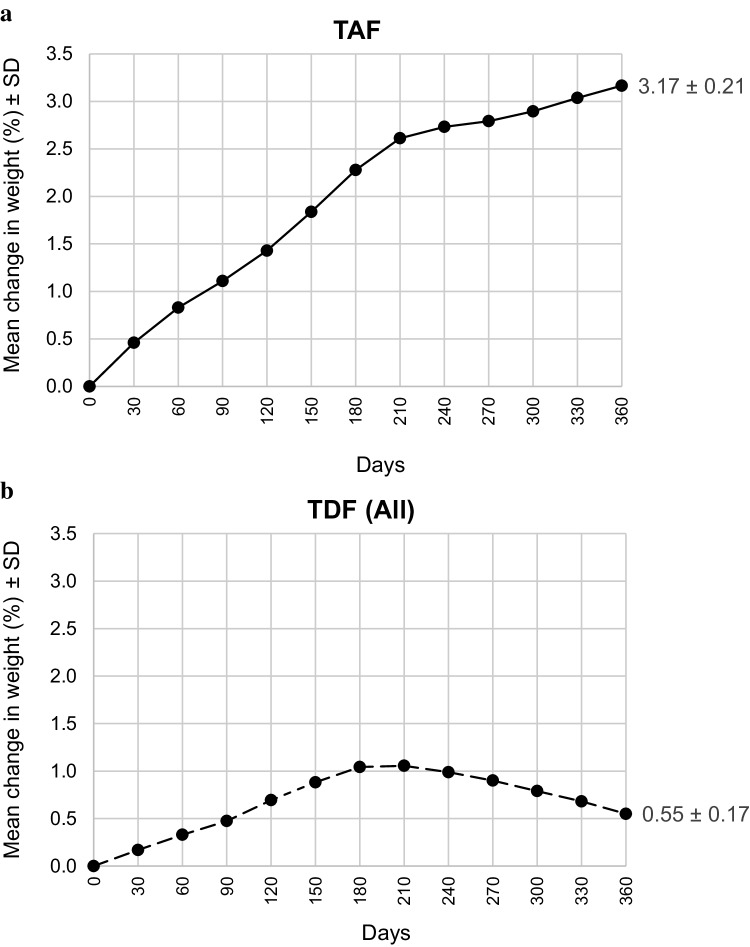
Mean change in weight in percent ± standard deviation through 360 days for TDF and TAF separately; **a** results of patients after switch to TAF (*n* = 129) and **b** results of patients receiving TDF; this arm comprises 241 patients, since switch patients initially received TDF (pooled data from the switch group and control group)

